# Comparing the efficacy of coefficient of variation control charts using generalized multiple dependent state sampling with various run-rule control charts

**DOI:** 10.1038/s41598-024-53296-6

**Published:** 2024-02-01

**Authors:** G. Srinivasa Rao, Muhammad Aslam, Faten S. Alamri, Chi-Hyuck Jun

**Affiliations:** 1 Department of Mathematics and Statistics, CNMS, The University of Dodoma, PO. Box: 259, Dodoma, Tanzania; 2 Department of Statistics, Faculty of Science, King Abdulaziz University, 21551 Jeddah, Saudi Arabia; 3 Department of Mathematical Sciences, College of Science, Princess Nourah Bint Abdulrahman University, P.O. Box 84428, 11671 Riyadh, Saudi Arabia; 4 Department of Industrial and Management Engineering, POSTECH, Pohang, Republic of Korea

**Keywords:** Engineering, Mathematics and computing

## Abstract

This paper aimed to develop a coefficient of variation (CV) control chart utilizing the generalized multiple dependent state (GMDS) sampling approach for CV monitoring. We conducted a comprehensive examination of this designed control chart in comparison to existing control charts based on multiple dependent state sampling (MDS) and the Shewhart-type CV control chart, with a focus on average run lengths. The results were then compared to run-rule control charts available in the existing literature. Additionally, we elucidated the implementation of the proposed control chart through concrete examples and a simulation study. The findings clearly demonstrated that the GMDS sampling control chart shows significantly superior accuracy in detecting process shifts when compared to the MDS sampling control chart. As a result, the control chart approach presented in this paper holds significant potential for applications in textile and medical industries, particularly when researchers seek to identify minor to moderate shifts in the CV, contributing to enhanced quality control and process monitoring in these domains.

## Introduction

For most of the industrial production process, the variation in the production process is due to two causes of fluctuation namely, the chance reason for the discrepancy and assignable reason for the fluctuation. Since Shewhart introduced control chart procedures to monitor the different manufacturing processes Shewhart control charts are more extensively used in most of the manufacturing industries and they are conformed to an effective tool to detect large shifts rapidly^1^.

The CV control chart is intended to inspect the method when the mean and standard deviation (SD) become shaky. Conventionally, the Shewhart control charts in support of variables are used to monitor mean and variance. To examine the process mean a Shewhart mean ($\overline{X}$) control chart may be utilized, whereas, for monitoring the process variance Shewhart range (*R*) or SD (S) or Variance ($S^{2}$) control charts. Hence, to examine the process mean and variance at the same time, single chart is studied by many authors, more details can be seen in Costa^2^.

Apart from this, Reed et al.^3^ rightly pointed out that, to monitor the quality control in chemical and biological experimentation and^4,5^ stated that CV chart has more application in the field of chemicals and metal manufacturing industries. Nevertheless, for some manufacturing processes the ratio of standard deviation and mean remains constant. Therefore, a statistic CV can be defined as the proportion of SD to mean, which means, CV, $\gamma = \frac{\sigma }{\mu }$. Kang et al. suggested a CV chart for examining the production process^6^. For more applications and study on control charts for CV one can refer to^7–25^.

Several researchers developed diversified sampling designs to get better efficiency towards control charts. Recently, more researchers focused on multiple dependent state (MDS) sampling in designing a new control chart. In this scheme, to decide about the state of the process the previous subgroup information is used in addition to the current subgroup information. Wortham and Baker proposed the MDS sampling in quality control charts^26^. MDS design is more efficient than the existing single sampling plans due to considering the previous lot along with the current lot consideration to make a decision. For more details, see Balamurali and Jun^27^. Vaerst developed a procedure to construct multiple deferred state sampling plan^28^. Soundararajan and Vijayaraghavan studied the designing multiple deferred state sampling plans involving minimum risks^29^. Soundararajan and Vijayaraghavan developed the procedure to construction and selection of multiple dependent (deferred) state sampling plan^30^. Later on Kuralmani and Govlndaraju studied multiple deferred (dependent) state sampling plans for given acceptable quality level and limiting quality level^31,32^. Aslam with various authors developed numerous articles using multiple dependent state sampling for various distributions refer^33–38^. Khan et al. developed a new X-bar control chart for multiple dependent state sampling using neutrosophic exponentially weighted moving average statistics with application to monitoring road accidents and road injuries^39^. Saghir et al. studied monitoring process variation using modified EWMA^40^. Balamurali and Jeyadurga attempted an attribute np control chart for monitoring mean life using multiple deferred state sampling based on truncated life tests^41^. Noor-ul-Amin and Riaz considered EWMA control chart for coefficient of variation using log-normal transformation under ranked set sampling^42^. Nguyen et al. studied the performance of VSI Shewhart control chart for monitoring the coefficient of variation in the presence of measurement errors^43^. Zamanzade Al-Omari considered a new ranked set sampling for estimating the population mean and variance^44^. Nawaz and Han developed a control chart to monitoring the process location by using new ranked set sampling-based memory type^45^.

More recently, designing the control charts using generalized multiple dependent state sampling for various environments have been studied by various authors for more details see^46–51^. The chief advantage of GMDS schemes over MDS schemes is flexibility in GMDS scheme, by using only *k* out of *m* previous values instead of the whole previous *m* values. It is important to highlight that, in simple random sample (SRS); decisions regarding the process are made solely based on information from a single sample. In contrast, MDS and GMDS consider the previous *m* or *k* out of m subgroups in the in-control area when faced with indecision, contributing to the decision-making process**.** Soundararajan and Vijayaraghavan^30^ considered a new attribute control chart using multiple dependent state sampling, Yan et al.^16^ studied designing a multiple state repetitive group sampling plan based on the coefficient of variation and^46^ deliberated a new variable control chart under generalized multiple dependent state sampling. Whereas, in this study, we considered the design of coefficient of variation control chart using GMDS sampling. Woodall et al.^52^ argued that the MDS and GMDS are equivalent to run-rule control chart. Muñoz et al.^53^ showed that the performance of run-rule control charts can be improved if the initial state is designed by MDS or GMDS.

This article is primarily motivated by the need to create a specialized control chart aimed at monitoring and assessing the variability within a given process. In particular, this control chart is centered on the Coefficient of Variation (CV), a critical statistical measure used to evaluate the relative variability of a dataset. To achieve this, the study introduces the concept of a control chart that operates in tandem with the Generalized Multiple Dependent State (GMDS) sampling scheme, which has not been extensively explored in the existing literature. One notable aspect of this research is the apparent gap in the current body of knowledge. Through a thorough review of existing literature, it becomes evident that there is a lack of comprehensive research that has proposed or examined the use of CV control charts in conjunction with the GMDS sampling design. This gap underscores the novelty and significance of the present study, as it not only seeks to fill this void in the research landscape but also aims to offer a robust and effective solution for monitoring and maintaining process quality and control, especially in situations where even minor to moderate shifts in the coefficient of variation can have substantial implications. The remaining paper can be organized as follows. The design of the CV control chart ($\gamma$-control chart) with reference to GMDS sampling is given in "Design of γ-control chart based on GMDS sampling" section. The tables of chart constants along with out-of-control ARLs are conducted in "Results and discussion" section. The sensitivity of the designed control chart over existing control charts are contributed in "Comparative studies" section. Also, methodology described using real practical data and a simulation analysis is also provided in "Comparative studies"section. In the end, some closing comments are provided in "Conclusions" section.

## Design of $\gamma$-control chart based on GMDS sampling

Let us assume that $Y_{1} ,Y_{2} ,...,Y_{n}$ be an independent values of the normal population with process mean $\mu$ and SD, $\sigma$. The theoretical CV ($\gamma$) is denoted by $\gamma = \frac{\sigma }{\mu }$. The observed CV ($\hat{\gamma }$) is given by $\hat{\gamma } = \frac{{S_{Y} }}{{\overline{Y}}}$. Where, $\overline{Y} = \frac{1}{n}\sum\nolimits_{i = 1}^{n} {Y_{i} \;{\text{and}}\;S_{Y} = \sqrt {\frac{1}{n - 1}\sum\nolimits_{i = 1}^{n} {\left( {Y_{i} - \overline{Y}} \right)^{2} } } }$.

According to^54^, if the measurement of characteristic follows $N\left( {\mu ,\sigma^{2} } \right)$ then the term $\frac{\sqrt n }{{\hat{\gamma }}}$ is follows as $t_{n - 1,\delta }$ where $t_{n - 1,\delta }$ is a non-central $t$ distribution with $\left( {n - 1} \right)$ degrees of freedom (d. f.) and non-central constraint $\delta = \frac{\sqrt n }{\gamma }$. Hence, the distribution function of $\hat{\gamma }$ is obtained as:

1 $$ G_{{\hat{\gamma }}} \left( {x|n,\gamma } \right) = 1 - G_{t} \left( {\frac{\sqrt n }{x}|n - 1,\frac{\sqrt n }{\gamma }} \right), $$

where $G_{t} (.)$ is the distribution function of the non-central $t$ distribution.

The designed $\gamma$-control chart with reference to GMDS sampling consists of the following inner and outer control limits:

Inner control limits are

$${\text{UCL}}_{2} = \mu_{0} \left( {\hat{\gamma }} \right) + k_{2} \,\sigma_{0} \left( {\hat{\gamma }} \right)$$

and

2 $$ {\text{LCL}}_{2} = \mu_{0} \left( {\hat{\gamma }} \right) - k_{2} \,\sigma_{0} \left( {\hat{\gamma }} \right) $$

Outer control limits are

$${\text{UCL}}_{1} = \mu_{0} \left( {\hat{\gamma }} \right) + k_{1} \,\sigma_{0} \left( {\hat{\gamma }} \right)$$

and

3 $$ {\text{LCL}}_{1} = \mu_{0} \left( {\hat{\gamma }} \right) - k_{1} \,\sigma_{0} \left( {\hat{\gamma }} \right) $$

where $k_{1}$ and $k_{2}$ are the chart constants to be obtained, $\mu_{0} \left( {\hat{\gamma }} \right)$ and $\sigma_{0} \left( {\hat{\gamma }} \right)$ are respectively the mean and standard deviation of sample CV $\hat{\gamma }$ when the process is under control. We use the following approximations for $\mu_{0} \left( {\hat{\gamma }} \right)$ and $\sigma_{0} \left( {\hat{\gamma }} \right)$ proposed by^54^ and used by^55,56^.

4 $$ \mu_{0} \left( {\hat{\gamma }} \right) \approx \gamma_{0} \left[ {1 + \frac{1}{n}\left( {\gamma_{0}^{2} - \frac{1}{4}} \right) + \frac{1}{{n^{2} }}\left( {3\gamma_{0}^{4} - \frac{{\gamma_{0}^{2} }}{4} - \frac{7}{12}} \right) + \frac{1}{{n^{3} }}\left( {15\gamma_{0}^{6} - \frac{{3\gamma_{0}^{4} }}{4} - \frac{{7\gamma_{0}^{2} }}{32} - \frac{19}{{128}}} \right)} \right] $$

5 $$ \sigma_{0} \left( {\hat{\gamma }} \right) \approx \gamma_{0} \left[ {\frac{1}{n}\left( {\gamma_{0}^{2} + \frac{1}{2}} \right) + \frac{1}{{n^{2} }}\left( {8\gamma_{0}^{4} + \gamma_{0}^{2} + \frac{3}{8}} \right) + \frac{1}{{n^{3} }}\left( {69\gamma_{0}^{6} + \frac{{7\gamma_{0}^{4} }}{2} + \frac{{3\gamma_{0}^{2} }}{4} + \frac{3}{16}} \right)} \right]^{{{1 \mathord{\left/ {\vphantom {1 2}} \right. \kern-0pt} 2}}} $$

where $\gamma_{0}$ is observed CV when the process under control.

The designed $\gamma$-control chart with reference to the GMDS scheme is functioning as the following steps:

1. Calculate $\hat{\gamma }$ from the observed sample values of size *n*.
2. The manufactured output is said to be under control if $LCL_{2} \le \hat{\gamma } \le UCL_{2}$ and the manufactured output is declared as out-of-control if $\hat{\gamma } \ge UCL_{1} \,\,{\text{or}}\,\hat{\gamma } \le LCL_{1}$.Otherwise, go to Step3.
3. The manufactured output is declared as under control whenever *k* (*k* ≤ m) out of m proceeding $\hat{\gamma }$ terms fall within $LCL_{2} \le \hat{\gamma } \le UCL_{2}$, otherwise, the manufactured output is considered as out of control.

The probability that the phenomenon is under control with reference to GMDS sampling scheme^47^ is given below:

6 $$ P_{in.0} = P_{a.0} + P_{s.0} \left[ {\sum\limits_{j = k}^{m} {\left( \begin{gathered} m \hfill \\ j \hfill \\ \end{gathered} \right)P_{a.0}^{j} \left( {1 - P_{a.0} } \right)^{m - j} } } \right] $$

where

7 $$ \begin{aligned} P_{a.0} & = p\left( {LCL_{2} \le \hat{\gamma } \le UCL_{2} |\gamma = \gamma_{0} } \right) \\ & = p\left( {\hat{\gamma } \le UCL_{2} |\gamma = \gamma_{0} } \right) - p\left( {\hat{\gamma } \le LCL_{2} |\gamma = \gamma_{0} } \right) \\ & = G_{{\hat{\gamma }}} \left( {UCL_{2} |\gamma = \gamma_{0} } \right) - G_{{\hat{\gamma }}} \left( {LCL_{2} |\gamma = \gamma_{0} } \right) \\ & = 1 - G_{t} \left[ {{{\sqrt n } \mathord{\left/ {\vphantom {{\sqrt n } {UCL_{2} |n - 1,{{\sqrt n } \mathord{\left/ {\vphantom {{\sqrt n } {\gamma_{0} }}} \right. \kern-0pt} {\gamma_{0} }}}}} \right. \kern-0pt} {UCL_{2} |n - 1,{{\sqrt n } \mathord{\left/ {\vphantom {{\sqrt n } {\gamma_{0} }}} \right. \kern-0pt} {\gamma_{0} }}}}} \right] - \left( {1 - G_{t} \left[ {{{\sqrt n } \mathord{\left/ {\vphantom {{\sqrt n } {LCL_{2} |n - 1,{{\sqrt n } \mathord{\left/ {\vphantom {{\sqrt n } {\gamma_{0} }}} \right. \kern-0pt} {\gamma_{0} }}}}} \right. \kern-0pt} {LCL_{2} |n - 1,{{\sqrt n } \mathord{\left/ {\vphantom {{\sqrt n } {\gamma_{0} }}} \right. \kern-0pt} {\gamma_{0} }}}}} \right]} \right) \\ & = G_{t} \left[ {{{\sqrt n } \mathord{\left/ {\vphantom {{\sqrt n } {LCL_{2} |n - 1,{{\sqrt n } \mathord{\left/ {\vphantom {{\sqrt n } {\gamma_{0} }}} \right. \kern-0pt} {\gamma_{0} }}}}} \right. \kern-0pt} {LCL_{2} |n - 1,{{\sqrt n } \mathord{\left/ {\vphantom {{\sqrt n } {\gamma_{0} }}} \right. \kern-0pt} {\gamma_{0} }}}}} \right] - G_{t} \left[ {{{\sqrt n } \mathord{\left/ {\vphantom {{\sqrt n } {UCL_{2} |n - 1,{{\sqrt n } \mathord{\left/ {\vphantom {{\sqrt n } {\gamma_{0} }}} \right. \kern-0pt} {\gamma_{0} }}}}} \right. \kern-0pt} {UCL_{2} |n - 1,{{\sqrt n } \mathord{\left/ {\vphantom {{\sqrt n } {\gamma_{0} }}} \right. \kern-0pt} {\gamma_{0} }}}}} \right] \\ \end{aligned} $$

and

8 $$ \begin{aligned} P_{s.0} & = p\left( {LCL_{1} \le \hat{\gamma } \le LCL_{2} |\gamma = \gamma_{0} } \right) + p\left( {UCL_{2} \le \hat{\gamma } \le UCL_{1} |\gamma = \gamma_{0} } \right) \\ & = G_{t} \left[ {{{\sqrt n } \mathord{\left/ {\vphantom {{\sqrt n } {LCL_{1} |n - 1,{{\sqrt n } \mathord{\left/ {\vphantom {{\sqrt n } {\gamma_{0} }}} \right. \kern-0pt} {\gamma_{0} }}}}} \right. \kern-0pt} {LCL_{1} |n - 1,{{\sqrt n } \mathord{\left/ {\vphantom {{\sqrt n } {\gamma_{0} }}} \right. \kern-0pt} {\gamma_{0} }}}}} \right] - G_{t} \left[ {{{\sqrt n } \mathord{\left/ {\vphantom {{\sqrt n } {LCL_{2} |n - 1,{{\sqrt n } \mathord{\left/ {\vphantom {{\sqrt n } {\gamma_{0} }}} \right. \kern-0pt} {\gamma_{0} }}}}} \right. \kern-0pt} {LCL_{2} |n - 1,{{\sqrt n } \mathord{\left/ {\vphantom {{\sqrt n } {\gamma_{0} }}} \right. \kern-0pt} {\gamma_{0} }}}}} \right] \\ & \quad + G_{t} \left[ {{{\sqrt n } \mathord{\left/ {\vphantom {{\sqrt n } {UCL_{2} |n - 1,{{\sqrt n } \mathord{\left/ {\vphantom {{\sqrt n } {\gamma_{0} }}} \right. \kern-0pt} {\gamma_{0} }}}}} \right. \kern-0pt} {UCL_{2} |n - 1,{{\sqrt n } \mathord{\left/ {\vphantom {{\sqrt n } {\gamma_{0} }}} \right. \kern-0pt} {\gamma_{0} }}}}} \right] - G_{t} \left[ {{{\sqrt n } \mathord{\left/ {\vphantom {{\sqrt n } {UCL_{1} |n - 1,{{\sqrt n } \mathord{\left/ {\vphantom {{\sqrt n } {\gamma_{0} }}} \right. \kern-0pt} {\gamma_{0} }}}}} \right. \kern-0pt} {UCL_{1} |n - 1,{{\sqrt n } \mathord{\left/ {\vphantom {{\sqrt n } {\gamma_{0} }}} \right. \kern-0pt} {\gamma_{0} }}}}} \right]. \\ \end{aligned} $$

Note that Eq. (6) aligns with the GMDS process, where, in the event of indecision, consideration is given to the previous *k* out of *m* subgroups in the in-control area. Consequently, the in-control decision relies on subgroups in both areas (indecision and in-control). As a result, Eq. (6) is formulated based on the binomial distribution.

Therefore, the average run length (ARL) when the manufactured output is under control is yielded below:

9 $$ ARL_{0} = \frac{1}{{1 - P_{in.0} }}. $$

Assume now that the relative variance has changed from $\gamma = \gamma_{0}$ to $\gamma = \gamma_{1} = c\gamma_{0}$, where *c* is a shift value when variance becomes the same. Thus, the statistic $\frac{\sqrt n }{{\hat{\gamma }_{1} }}$ is follows to $t_{n - 1,\delta }$ with $t_{n - 1,\delta }$ is a non-central $t$ distribution with the same d.f. whereas and non-centrality parameter constraint, $\delta = \frac{\sqrt n }{{c\gamma_{0} }}$.

The probability of manufactured the output is in-control while the relative variance has changed can be obtained as follows.

10 $$ P_{in.1} = P_{a.1} + P_{s.1} \left[ {\sum\limits_{j = k}^{m} {\left( \begin{gathered} m \hfill \\ j \hfill \\ \end{gathered} \right)P_{a.1}^{j} \left( {1 - P_{a.1} } \right)^{m - j} } } \right], $$

where

11 $$ \begin{aligned} P_{a.1} & = p\left( {LCL_{2} \le \hat{\gamma } \le UCL_{2} |\gamma = \gamma_{1} } \right) \\ & = G_{t} \left[ {{{\sqrt n } \mathord{\left/ {\vphantom {{\sqrt n } {LCL_{2} |n - 1,{{\sqrt n } \mathord{\left/ {\vphantom {{\sqrt n } {c\gamma_{0} }}} \right. \kern-0pt} {c\gamma_{0} }}}}} \right. \kern-0pt} {LCL_{2} |n - 1,{{\sqrt n } \mathord{\left/ {\vphantom {{\sqrt n } {c\gamma_{0} }}} \right. \kern-0pt} {c\gamma_{0} }}}}} \right] - G_{t} \left[ {{{\sqrt n } \mathord{\left/ {\vphantom {{\sqrt n } {UCL_{2} |n - 1,{{\sqrt n } \mathord{\left/ {\vphantom {{\sqrt n } {c\gamma_{0} }}} \right. \kern-0pt} {c\gamma_{0} }}}}} \right. \kern-0pt} {UCL_{2} |n - 1,{{\sqrt n } \mathord{\left/ {\vphantom {{\sqrt n } {c\gamma_{0} }}} \right. \kern-0pt} {c\gamma_{0} }}}}} \right]. \\ \end{aligned} $$

and

12 $$ \begin{aligned} P_{s.1} & = p\left( {LCL_{1} \le \hat{\gamma } \le LCL_{2} |\gamma = \gamma_{1} } \right) + p\left( {UCL_{2} \le \hat{\gamma } \le UCL_{1} |\gamma = \gamma_{1} } \right) \\ & = G_{t} \left[ {{{\sqrt n } \mathord{\left/ {\vphantom {{\sqrt n } {LCL_{1} |n - 1,{{\sqrt n } \mathord{\left/ {\vphantom {{\sqrt n } {c\gamma_{0} }}} \right. \kern-0pt} {c\gamma_{0} }}}}} \right. \kern-0pt} {LCL_{1} |n - 1,{{\sqrt n } \mathord{\left/ {\vphantom {{\sqrt n } {c\gamma_{0} }}} \right. \kern-0pt} {c\gamma_{0} }}}}} \right] - G_{t} \left[ {{{\sqrt n } \mathord{\left/ {\vphantom {{\sqrt n } {LCL_{2} |n - 1,{{\sqrt n } \mathord{\left/ {\vphantom {{\sqrt n } {c\gamma_{0} }}} \right. \kern-0pt} {c\gamma_{0} }}}}} \right. \kern-0pt} {LCL_{2} |n - 1,{{\sqrt n } \mathord{\left/ {\vphantom {{\sqrt n } {c\gamma_{0} }}} \right. \kern-0pt} {c\gamma_{0} }}}}} \right] \\ & \quad + G_{t} \left[ {{{\sqrt n } \mathord{\left/ {\vphantom {{\sqrt n } {UCL_{2} |n - 1,{{\sqrt n } \mathord{\left/ {\vphantom {{\sqrt n } {c\gamma_{0} }}} \right. \kern-0pt} {c\gamma_{0} }}}}} \right. \kern-0pt} {UCL_{2} |n - 1,{{\sqrt n } \mathord{\left/ {\vphantom {{\sqrt n } {c\gamma_{0} }}} \right. \kern-0pt} {c\gamma_{0} }}}}} \right] - G_{t} \left[ {{{\sqrt n } \mathord{\left/ {\vphantom {{\sqrt n } {UCL_{1} |n - 1,{{\sqrt n } \mathord{\left/ {\vphantom {{\sqrt n } {c\gamma_{0} }}} \right. \kern-0pt} {c\gamma_{0} }}}}} \right. \kern-0pt} {UCL_{1} |n - 1,{{\sqrt n } \mathord{\left/ {\vphantom {{\sqrt n } {c\gamma_{0} }}} \right. \kern-0pt} {c\gamma_{0} }}}}} \right] \\ \end{aligned} $$

The average run length (ARL) when the manufactured output is out-of-control is given as

13 $$ ARL_{1} = \frac{1}{{1 - P_{in.1} }}. $$

The chart constants $k_{1}$ and $k_{2}$ for the designed chart as well as ARL_1_ can be computed with the following step by step procedure:

1. Choose the preferred in-control ARL to say *r*_0_.
2. Select the specified values of *n*, *m* and *k*.
3. Determine the value of $P_{in.0}$ and hence obtain ARL_0_ for different possible values of $k_{1}$ and $k_{2}$.
4. Obtain the estimates of chart constants $k_{1}$ and $k_{2}$ in such a way that $ARL_{0} \ge r_{0}$ based on 100,000 simulations.
5. In Step 4 will give more values of $k_{1}$ and $k_{2}$, select the specified estimate of $k_{1}$ and $k_{2}$ such that to minimum value of $ARL_{0}$.
6. By means of the determines of $k_{1}$ and $k_{2}$ obtained in Step 5, compute $P_{in.1}$ and hence acquire the $ARL_{1}$ and SD of run-length (SDRL) for distinct changed values.

## Results and discussion

The achievement of designed $\gamma$-control chart is studied based on ARL, both under control ARL (ARL_0_) and beyond control ARL (ARL_1_). To study the achievement of the control chart, we use to compare the ARL values. A control chart is considered a good control chart if the lower value of ARL_1_ and the larger value of ARL_0_. The designed chart based on two control chart constants namely $k_{1}$ and $k_{2}$ along with sample size *n*, *m*, *k*. In this paper, the Monte Carlo simulation is carried out to obtain the chart constants based on the algorithm given in "Design of γ-control chart based on GMDS sampling" section. An algorithm is developed in R language, 100,000 replications used to obtain chart constants and hence beyond control ARL and SDRL values for various changed values, *c* from 1.0 to 2.0 with an interval of 0.1 and 2.0 to 4.0 with an interval of 0.5. The results are computed for the sample size, *n* = 5 and 7; the values of *m* considered as 4 and 5; $\gamma_{0}$ = 0.05 and 0.20. Tables 1 and 2 are for $r_{0}$ = 370 and $\gamma_{0}$ = 0.05; Tables 3 and 4 are for $r_{0}$ = 370 and $\gamma_{0}$ = 0.20; and Tables 5 and 6 are for $r_{0}$ = 500 and $\gamma_{0}$ = 0.20.

**Table 1:** ARLs and SDRLs of $\gamma$-control chart for GMDSS when *n* = 5, $\gamma_{0}$ = 0.05, ARL_0_ = 370.

*c*	$k_{1}$ = 3.0335		$k_{1}$ = 4.0885		$k_{1}$ = 4.9205		$k_{1}$ = 3.255		$k_{1}$ = 4.1490		$k_{1}$ = 4.4695	
	$k_{2}$ = 1.6145		$k_{2}$ = 1.8205		$k_{2}$ = 2.3005		$k_{2}$ = 1.6105		$k_{2}$ = 1.8960		$k_{2}$ = 2.3445	
	*m* = 4,*k* = 2		*m* = 4,*k* = 3		*m* = 4,*k* = 4		*m* = 5,*k* = 3		*m* = 5,*k* = 4		*m* = 5,*k* = 5	
	ARL	SDRL	ARL	SDRL	ARL	SDRL	ARL	SDRL	ARL	SDRL	ARL	SDRL
1.0	370.13	369.63	370.00	369.50	370.02	369.52	370.09	369.59	370.15	369.65	370.14	369.64
1.1	86.85	86.34	100.82	100.32	101.60	101.10	86.27	85.77	94.60	94.10	97.52	97.02
1.2	27.44	26.93	39.88	39.38	36.41	35.90	28.94	28.44	36.18	35.68	32.90	32.40
1.3	13.32	12.81	15.85	15.34	15.32	14.81	12.76	12.25	14.13	13.62	13.93	13.42
1.4	7.55	7.03	8.01	7.49	8.15	7.63	6.83	6.31	7.16	6.64	7.52	7.00
1.5	4.85	4.32	4.85	4.32	5.13	4.60	4.27	3.74	4.40	3.87	4.82	4.30
1.6	3.44	2.90	3.36	2.82	3.65	3.11	3.02	2.47	3.11	2.56	3.49	2.95
1.7	2.65	2.09	2.57	2.01	2.83	2.28	2.34	1.78	2.42	1.85	2.76	2.20
1.8	2.16	1.58	2.10	1.52	2.34	1.77	1.95	1.36	2.02	1.43	2.31	1.73
1.9	1.85	1.26	1.81	1.22	2.02	1.44	1.69	1.08	1.77	1.16	2.01	1.42
2.0	1.64	1.03	1.62	1.01	1.80	1.20	1.53	0.90	1.60	0.98	1.80	1.21
2.5	1.22	0.51	1.23	0.53	1.32	0.65	1.19	0.48	1.24	0.54	1.33	0.67
3.0	1.10	0.33	1.11	0.36	1.16	0.44	1.10	0.32	1.12	0.37	1.17	0.45
3.5	1.06	0.24	1.06	0.26	1.09	0.32	1.05	0.24	1.07	0.27	1.10	0.33
4.0	1.03	0.19	1.04	0.20	1.06	0.25	1.03	0.18	1.04	0.21	1.06	0.25

**Table 2:** ARLs and SDRLs of $\gamma$-control chart for GMDSS when *n* = 7, $\gamma_{0}$ = 0.05, ARL_0_ = 370.

*c*	$k_{1}$ = 3.1560		$k_{1}$ = 3.1105		$k_{1}$ = 3.1115		$k_{1}$ = 3.3105		$k_{1}$ = 4.1225		$k_{1}$ = 4.3435	
	$k_{2}$ = 1.6110		$k_{2}$ = 2.1175		$k_{2}$ = 2.6305		$k_{2}$ = 1.6105		$k_{2}$ = 1.8715		$k_{2}$ = 2.3145	
	*m* = 4,*k* = 2		*m* = 4,*k* = 3		*m* = 4,*k* = 4		*m* = 5,*k* = 3		*m* = 5,*k* = 4		*m* = 5,*k* = 5	
	ARL	SDRL	ARL	SDRL	ARL	SDRL	ARL	SDRL	ARL	SDRL	ARL	SDRL
1.0	370.10	369.60	370.10	369.60	370.08	369.58	370.09	369.59	370.10	369.60	370.13	369.63
1.1	94.74	94.23	96.43	95.92	97.84	97.34	82.51	81.01	83.47	82.96	86.26	85.76
1.2	26.95	26.45	27.70	27.19	29.29	28.79	20.31	19.81	21.26	20.75	23.20	22.69
1.3	10.32	9.81	10.65	10.14	11.87	11.36	7.26	6.74	7.91	7.39	9.04	8.52
1.4	5.17	4.64	5.37	4.84	6.23	5.71	3.48	3.24	4.09	3.55	4.79	4.26
1.5	3.52	2.98	3.62	3.08	4.19	3.65	2.86	2.31	2.94	2.38	3.39	2.84
1.6	2.48	1.91	2.59	2.03	3.05	2.50	2.04	1.26	2.17	1.59	2.53	1.96
1.7	1.93	1.34	2.04	1.46	2.41	1.84	1.72	1.11	1.77	1.17	2.05	1.47
1.8	1.61	1.00	1.72	1.12	2.02	1.43	1.49	0.85	1.54	0.92	1.77	1.16
1.9	1.43	0.78	1.53	0.90	1.76	1.16	1.35	0.68	1.40	0.75	1.58	0.95
2.0	1.31	0.63	1.40	0.74	1.58	0.96	1.26	0.57	1.31	0.63	1.45	0.80
2.5	1.08	0.30	1.13	0.38	1.20	0.48	1.08	0.29	1.10	0.33	1.15	0.42
3.0	1.03	0.18	1.05	0.23	1.08	0.30	1.03	0.18	1.04	0.21	1.06	0.26
3.5	1.01	0.12	1.02	0.16	1.04	0.20	1.01	0.12	1.02	0.14	1.03	0.17
4.0	1.01	0.08	1.01	0.11	1.02	0.14	1.01	0.08	1.01	0.10	1.01	0.12

**Table 3:** ARLs and SDRLs of $\gamma$-control chart for GMDSS when *n* = 5, $\gamma_{0}$ = 0.20, ARL_0_ = 370.

*c*	$k_{1}$ = 3.4205		$k_{1}$ = 3.3735		$k_{1}$ = 3.3675		$k_{1}$ = 3.5225		$k_{1}$ = 3.3945		$k_{1}$ = 3.3935	
	$k_{2}$ = 1.6155		$k_{2}$ = 2.2395		$k_{2}$ = 3.0595		$k_{2}$ = 1.6115		$k_{2}$ = 2.1785		$k_{2}$ = 2.8555	
	*m* = 4,*k* = 2		*m* = 4,*k* = 3		*m* = 4,*k* = 4		*m* = 5,*k* = 3		*m* = 5,*k* = 4		*m* = 5,*k* = 5	
	ARL SDRL		ARL	SDRL	ARL	SDRL	ARL	SDRL	ARL	SDRL	ARL	SDRL
1.0	370.05	369.55	370.13	369.63	370.11	369.61	370.05	369.55	370.11	369.61	370.13	369.63
1.1	103.64	103.14	104.15	103.65	107.42	106.92	97.50	96.99	98.58	98.08	101.94	101.44
1.2	38.43	37.93	39.05	38.55	42.33	41.83	33.56	33.06	35.01	34.51	38.38	37.87
1.3	17.61	17.10	18.07	17.56	20.73	20.22	14.59	14.08	15.63	15.12	18.23	17.72
1.4	9.54	9.03	9.88	9.37	11.92	11.41	7.72	7.20	8.44	7.92	10.33	9.82
1.5	5.92	5.39	6.18	5.66	7.74	7.22	4.78	4.26	5.30	4.78	6.70	6.18
1.6	4.08	3.55	4.30	3.77	5.51	4.98	3.35	2.80	3.75	3.21	4.81	4.28
1.7	3.07	2.52	3.26	2.71	4.21	3.68	2.57	2.01	2.89	2.34	3.72	3.18
1.8	2.46	1.89	2.63	2.07	3.40	2.85	2.11	1.53	2.38	1.81	3.04	2.49
1.9	2.07	1.49	2.23	1.65	2.86	2.30	1.82	1.22	2.06	1.47	2.59	2.03
2.0	1.81	1.21	1.95	1.37	2.48	1.92	1.63	1.01	1.83	1.24	2.27	1.70
2.5	1.29	0.61	1.38	0.73	1.63	1.01	1.24	0.55	1.35	0.69	1.55	0.93
3.0	1.14	0.40	1.21	0.50	1.34	0.68	1.13	0.38	1.19	0.48	1.30	0.63
3.5	1.08	0.30	1.13	0.38	1.22	0.51	1.08	0.29	1.12	0.37	1.19	0.48
4.0	1.06	0.24	1.09	0.31	1.15	0.41	1.05	0.24	1.08	0.30	1.13	0.38

**Table 4:** ARLs and SDRLs of $\gamma$-control chart for GMDSS when *n* = 7, $\gamma_{0}$ = 0.20, ARL_0_ = 370.

*c*	$k_{1}$ = 3.3085		$k_{1}$ = 3.2525		$k_{1}$ = 3.2495		$k_{1}$ = 3.4215		$k_{1}$ = 3.2735		$k_{1}$ = 3.2695	
	$k_{2}$ = 1.6155		$k_{2}$ = 2.2675		$k_{2}$ = 2.9675		$k_{2}$ = 1.6115		$k_{2}$ = 2.1855		$k_{2}$ = 2.7975	
	*m* = 4,*k* = 2		*m* = 4,*k* = 3		*m* = 4,*k* = 4		*m* = 5,*k* = 3		*m* = 5,*k* = 4		*m* = 5,*k* = 5	
	ARL	SDRL	ARL	SDRL	ARL	SDRL	ARL	SDRL	ARL	SDRL	ARL	SDRL
1.0	370.05	369.55	370.15	369.65	370.14	369.64	370.10	369.60	370.07	369.57	370.15	369.64
1.1	89.32	88.82	90.31	89.80	92.20	91.70	84.44	83.94	85.18	84.68	87.49	86.99
1.2	29.42	28.91	30.74	30.23	32.92	32.42	25.38	24.87	27.16	26.65	29.81	29.31
1.3	12.39	11.88	13.32	12.81	15.18	14.67	10.10	9.59	11.31	10.80	13.36	12.85
1.4	6.41	5.89	7.02	6.50	8.45	7.94	5.16	4.63	5.92	5.40	7.39	6.87
1.5	3.92	3.39	4.35	3.82	5.43	4.90	3.21	2.66	3.73	3.19	4.78	4.25
1.6	2.74	2.18	3.06	2.51	3.88	3.34	2.31	1.74	2.69	2.13	3.46	2.92
1.7	2.11	1.53	2.36	1.79	3.00	2.45	1.84	1.25	2.14	1.56	2.72	2.16
1.8	1.75	1.15	1.96	1.37	2.46	1.89	1.58	0.96	1.82	1.22	2.26	1.69
1.9	1.53	0.90	1.70	1.09	2.10	1.52	1.42	0.77	1.61	0.99	1.96	1.37
2.0	1.39	0.73	1.54	0.91	1.86	1.26	1.31	0.64	1.47	0.84	1.75	1.15
2.5	1.12	0.36	1.19	0.48	1.32	0.65	1.11	0.34	1.18	0.45	1.28	0.60
3.0	1.05	0.23	1.09	0.31	1.15	0.42	1.05	0.23	1.08	0.30	1.13	0.39
3.5	1.03	0.16	1.05	0.22	1.08	0.30	1.03	0.16	1.04	0.22	1.07	0.28
4.0	1.02	0.12	1.03	0.17	1.05	0.23	1.02	0.12	1.03	0.16	1.04	0.21

**Table 5:** ARLs and SDRLs of $\gamma$-control chart for GMDSS when *n* = 5 $\gamma_{0}$ = 0.20, ARL_0_ = 500.

c	$k_{1}$ = 3.5045		$k_{1}$ = 3.5015		$k_{1}$ = 3.5005		$k_{1}$ = 3.5045		$k_{1}$ = 3.5025		$k_{1}$ = 3.5025	
	$k_{2}$ = 1.9405		$k_{2}$ = 2.6845		$k_{2}$ = 3.4535		$k_{2}$ = 2.0395		$k_{2}$ = 2.6725		$k_{2}$ = 3.3555	
	*m* = 4,*k* = 2		*m* = 4,*k* = 3		*m* = 4,*k* = 4		*m* = 5,*k* = 3		*m* = 5,*k* = 4		*m* = 5,*k* = 5	
	ARL	SDRL	ARL	SDRL	ARL	SDRL	ARL	SDRL	ARL	SDRL	ARL	SDRL
1.0	500.07	499.57	500.11	499.61	500.00	499.50	500.04	499.54	500.06	499.56	500.14	499.63
1.1	138.03	137.53	139.25	138.75	140.40	139.90	137.10	136.60	138.21	137.71	139.44	138.94
1.2	51.76	51.25	52.96	52.46	54.48	53.98	50.54	50.04	51.80	51.30	53.58	53.08
1.3	23.91	23.41	24.92	24.41	26.49	25.98	22.77	22.26	23.90	23.40	25.77	25.27
1.4	12.88	12.37	13.68	13.17	15.14	14.63	11.96	11.45	12.89	12.38	14.59	14.08
1.5	7.84	7.32	8.46	7.94	9.75	9.23	7.15	6.63	7.88	7.37	9.33	8.82
1.6	5.27	4.74	5.76	5.23	6.86	6.34	4.76	4.23	5.35	4.82	6.55	6.02
1.7	3.84	3.30	4.24	3.70	5.17	4.64	3.48	2.93	3.95	3.41	4.93	4.40
1.8	2.99	2.44	3.32	2.78	4.11	3.57	2.72	2.17	3.12	2.57	3.92	3.38
1.9	2.45	1.89	2.73	2.18	3.40	2.86	2.26	1.68	2.59	2.03	3.25	2.71
2.0	2.10	1.52	2.34	1.77	2.91	2.35	1.95	1.36	2.24	1.66	2.79	2.24
2.5	1.38	0.72	1.52	0.89	1.80	1.20	1.35	0.69	1.50	0.87	1.75	1.15
3.0	1.19	0.47	1.28	0.60	1.43	0.79	1.18	0.46	1.27	0.59	1.41	0.76
3.5	1.11	0.35	1.17	0.45	1.27	0.58	1.11	0.35	1.17	0.45	1.25	0.56
4.0	1.07	0.28	1.12	0.36	1.18	0.47	1.08	0.28	1.12	0.36	1.17	0.45

**Table 6:** ARLs and SDRLs of $\gamma$-control chart for GMDSS when *n* = 7 $\gamma_{0}$ = 0.20, ARL_0_ = 500.

c	$k_{1}$ = 3.3755		$k_{1}$ = 3.3745		$k_{1}$ = 3.3745		$k_{1}$ = 3.3765		$k_{1}$ = 3.3755		$k_{1}$ = 3.3755	
	$k_{2}$ = 2.0785		$k_{2}$ = 2.8535		$k_{2}$ = 3.3505		$k_{2}$ = 2.1175		$k_{2}$ = 2.6805		$k_{2}$ = 3.2865	
	*m* = 4,*k* = 2		*m* = 4,*k* = 3		*m* = 4,*k* = 4		*m* = 5,*k* = 3		*m* = 5,*k* = 4		*m* = 5,*k* = 5	
	ARL	SDRL	ARL	SDRL	ARL	SDRL	ARL	SDRL	ARL	SDRL	ARL	SDRL
1.0	500.01	499.51	500.10	499.60	500.12	499.62	500.11	499.61	500.01	499.51	500.14	499.64
1.1	119.52	119.02	120.39	119.79	120.56	120.06	118.55	118.05	118.92	118.42	119.96	119.46
1.2	41.13	40.63	42.17	41.57	42.56	42.06	39.86	39.36	40.47	39.97	42.02	41.52
1.3	18.00	17.49	18.93	18.43	19.56	19.05	16.82	16.32	17.51	17.01	19.14	18.63
1.4	9.36	8.84	10.18	9.66	10.83	10.32	8.45	7.94	9.08	8.57	10.53	10.01
1.5	5.58	5.06	6.25	5.73	6.88	6.36	4.95	4.42	5.47	4.95	6.65	6.13
1.6	3.73	3.20	4.27	3.74	4.83	4.30	3.31	2.76	3.73	3.19	4.67	4.14
1.7	2.75	2.19	3.18	2.63	3.66	3.12	2.46	1.90	2.81	2.25	3.54	3.00
1.8	2.18	1.60	2.53	1.97	2.93	2.38	1.98	1.40	2.27	1.70	2.84	2.29
1.9	1.83	1.23	2.12	1.54	2.46	1.89	1.70	1.09	1.94	1.35	2.39	1.82
2.0	1.60	0.98	1.85	1.26	2.13	1.55	1.52	0.89	1.72	1.11	2.08	1.50
2.5	1.18	0.46	1.30	0.62	1.42	0.77	1.17	0.45	1.26	0.57	1.40	0.75
3.0	1.08	0.29	1.14	0.40	1.20	0.49	1.08	0.29	1.12	0.37	1.19	0.47
3.5	1.04	0.21	1.08	0.29	1.11	0.35	1.04	0.21	1.07	0.27	1.10	0.34
4.0	1.02	0.16	1.05	0.22	1.07	0.26	1.02	0.16	1.04	0.20	1.06	0.26

We noticed the following few significant remarks from Tables 1, 2, 3, 4, 5 and 6 for the planned control charts:

1. The beyond control ARL and SDRL values decrements rapidly while the process is a shift (${\text{c}}$) increments from 1.0 to 4.0.
2. It is observed from the output that lower trend in ARL_1_ and SDRL values when ${\text{n}}$ increments from 5 to 7 under the condition that the remaining parametric combinations are fixed.
3. Results discovered that ARL_1_ and SDRL quantities lower as *m* values increases. Further, identified as *k* increments the quantities of ARL_1_ and SDRL also increases. A similar tendency noticed for several sample sizes and various ${{\text{ARL}}}_{0}$ quantities.
4. Results show that, an increasing tendency for chart coefficient $k_{2}$ with an increasing value of *k* when *m* value is fixed.
5. The significance noticed from tables is that ARL_1_ and SDRL figures are diminutive for *k* = *m* − 2 as considered among quantities of *k* for the determined quantity of *m*. Moreover, observed that while *k* = *m* the quantity of ARL_1_ and SDRL is superior to at *k* = *m* − 1 and *k* = *m* *−* 2 (since when *k* = *m*, the designed plan becomes MDS scheme). Therefore, the results indicate as the GMDS sampling control chart is great extent accurate than the MDS sampling control chart to differentiating the process shift.

## Comparative studies

In this division, relative learning is prepared among the existing Shewhart type $\gamma$-control chart, MDS $\gamma$-control chart, SH-$\gamma$ chart developed by^6^, in the 2-out-of-3 Run Rules (denoted as $RR_{2,3} - \gamma \,{\text{chart}}$) suggested by^5^ and designed $\gamma$-control chart. Even our proposed control chart outperform the 3-out-of-4 and the 4-out-of-5 Run Rules (denoted as the RR_3,4_-$\gamma$ chart and RR_4,5_-$\gamma$ chart). In addition, the industrial application of planned control chart and its supremacy over available control chart schemes studied using real data set. Furthermore, the achievement of the planned control chart with respect to available control charts also evaluate with a simulation study. We know that a control chart is considered to a greater extent competent than other control charts when that chart gives lesser ARL estimates. Therefore, the sensitivity of the proposed control chart over available charts can be studied based on ARL estimates. Here we consider ARL_0_ = 370, *n* = 5, $\gamma_{0}$ = 0.10 and 0.20 to study the sensitivity available schemes alongside the designed $\gamma$-control chart based on MDS and GMDS in favor of different mean changes, which are given in Table 7.

**Table 7:** ARLs comparison of different $\gamma$-control chart when ${{\text{ARL}}}_{0}=370$,* n* = 5 and *m* = 5.

*c*	$\gamma_{0}$ = 0.10					$\gamma_{0}$ = 0.20				
	Proposed $k_{1}$ = 3.3990 $k_{2}$ = 1.6100	MDS $k_{1}$ = 3.2785 $k_{2}$ = 2.7085	Shewhart L = 3.2331	SH-$\gamma$ chart	$RR_{2,3} - \gamma$	Proposed $k_{1}$ = 3.5225 $k_{2}$ = 1.6115	MDS $k_{1}$ = 3.3935 $k_{2}$ = 2.8555	Shewhart L = 3.3590	SH-$\gamma$ chart	$RR_{2,3} - \gamma$
1.0	370.12	370.13	370.00	370.4	370.4	370.05	370.13	370.03	370.4	370.4
1.1	95.97	97.06	107.64	160.6	101.5	97.50	101.94	110.12	164.0	101.7
1.2	32.38	35.29	43.00	65.3	39.3	33.56	38.38	44.72	68.1	40.0
1.3	13.87	16.42	21.43	49.4	33.7	14.59	18.23	22.55	57.4	46.8
1.4	7.27	9.23	12.51	26.9	22.1	7.72	10.33	13.27	31.3	28.7
1.5	4.49	5.98	8.20	10.8	8.4	4.78	6.70	8.75	11.6	8.8
1.6	3.14	4.30	5.86	9.2	6.7	3.35	4.81	6.28	10.3	9.2
1.7	2.42	3.34	4.47	7.1	5.8	2.57	3.72	4.80	8.7	7.8
1.8	1.99	2.75	3.59	6.3	4.8	2.11	3.04	3.86	7.1	6.5
1.9	1.73	2.35	2.99	4.8	3.9	1.82	2.59	3.22	5.8	4.5
2.0	1.55	2.08	2.58	2.9	3.6	1.63	2.27	2.78	3.2	2.6
2.5	1.20	1.45	1.63	1.8	2.6	1.24	1.55	1.75	1.9	1.7
3.0	1.10	1.23	1.32	1.6	1.9	1.13	1.30	1.41	1.7	1.6
3.5	1.06	1.13	1.19	1.4	1.6	1.08	1.19	1.26	1.5	1.4
4.0	1.04	1.09	1.12	1.2	1.4	1.05	1.13	1.17	1.3	1.2

SH-$\gamma$ chart suggested by Kang et al.^6^; the 2-out-of-3 Run Rules (denoted as $RR_{2,3} - \gamma \,{\text{chart}}$) suggested by Castagliola et al.^5^.

**Figure 1:**
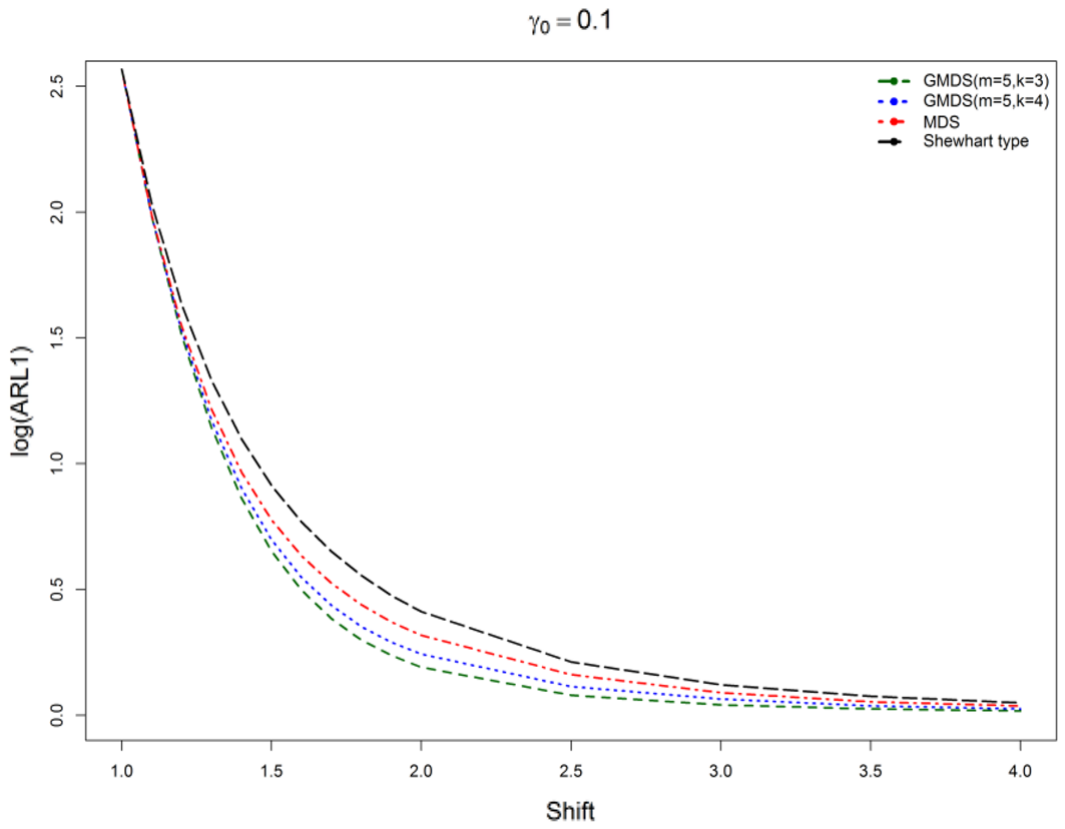
ARL curves of $\gamma$-control chart for three charts for *n* = 5, *m* = 5, $\gamma_{0}$ = 0.10, ARL_0_ = 370.

**Figure 2:**
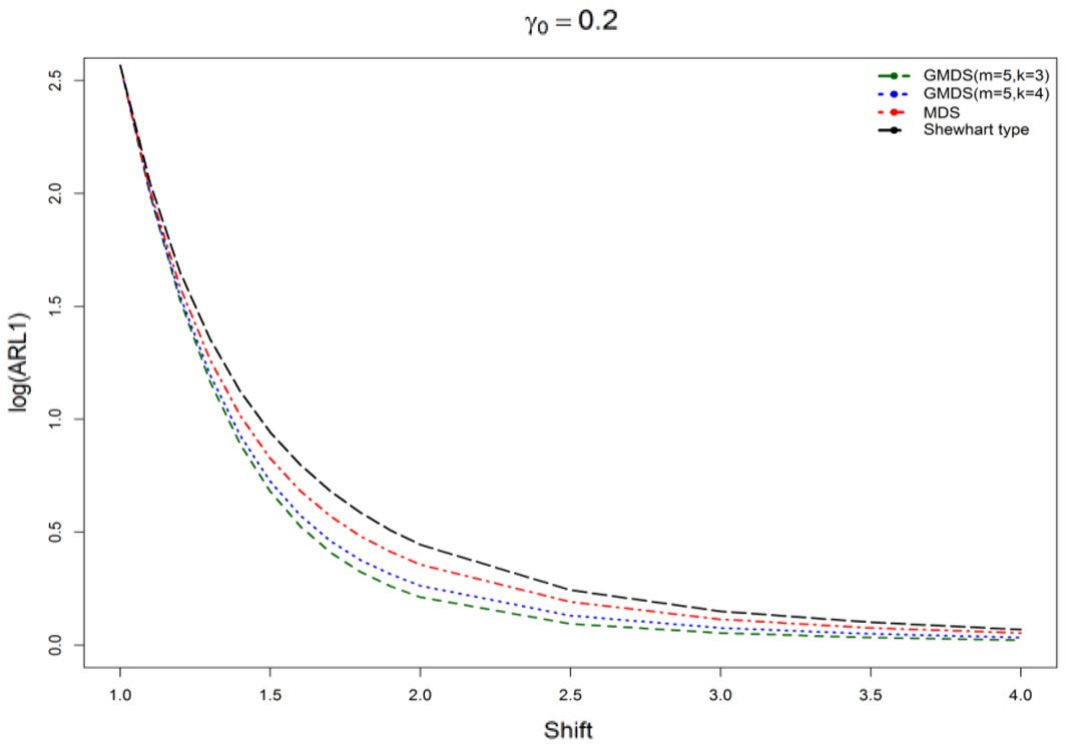
ARL curves of $\gamma$-control chart for three charts at *n* = 5, *m* = 5, $\gamma_{0}$ = 0.20, ARL_0_ = 370.

The results on the basis of Tables 7 revealed that planned control charts have fewer ARL_1_ values when compared with the MDS and Shewhart type control charts for different shifts (*c*) and various parametric combinations considered in this study. For example, if ARL_0_ = 370, *n* = 5, $\gamma_{0}$ = 0.10 and *c* = 1.5 on the basis of Tables 7, clear that the proposed control chart ARL_1_ = 4.49 on the other hand, ARL_1_ = 5.98 for MDS control chart, ARL_1_ = 8.20 for the Shewhart type control chart, ARL_1_ = 10.8 for SH-$\gamma$ chart and ARL_1_ = 8.4 for $RR_{2,3} - \gamma \,{\text{chart}}$. Similarly, for ARL_0_ = 370, *n* = 5, $\gamma_{0}$ = 0.20 and *c* = 1.2 from Tables 7, we pragmatic that the designed control chart as ARL_1_ = 33.56 although ARL_1_ = 38.38 for MDS control chart, ARL_1_ = 44.72 from the Shewhart type control chart, ARL_1_ = 68.1 for SH-$\gamma$ chart and ARL_1_ = 40.0 for $RR_{2,3} - \gamma \,{\text{chart}}$. Thus, the proposed control chart performed well as compared with the existing SH-$\gamma$ chart developed by^6^, the 2-out-of-3 Run Rules (denoted as $RR_{2,3} - \gamma \,{\text{chart}}$) suggested by^5^ control charts. Figures 1 and 2 depicted the ARL curves for designed $\gamma$-control chart for GMDS, MDS, and Shewhart type control charts for different shifts. Figures 1 and 2 show that it is clear that the proposed $\gamma$-control chart with reference to GMDS evidences a more sensitive than the MDS and the Shewhart type control charts. To accentuate these outcomes an actual data exemplar and a simulation study also conducted in the subsequent sub-sections.

### Industrial application-1

**Figure 3:**
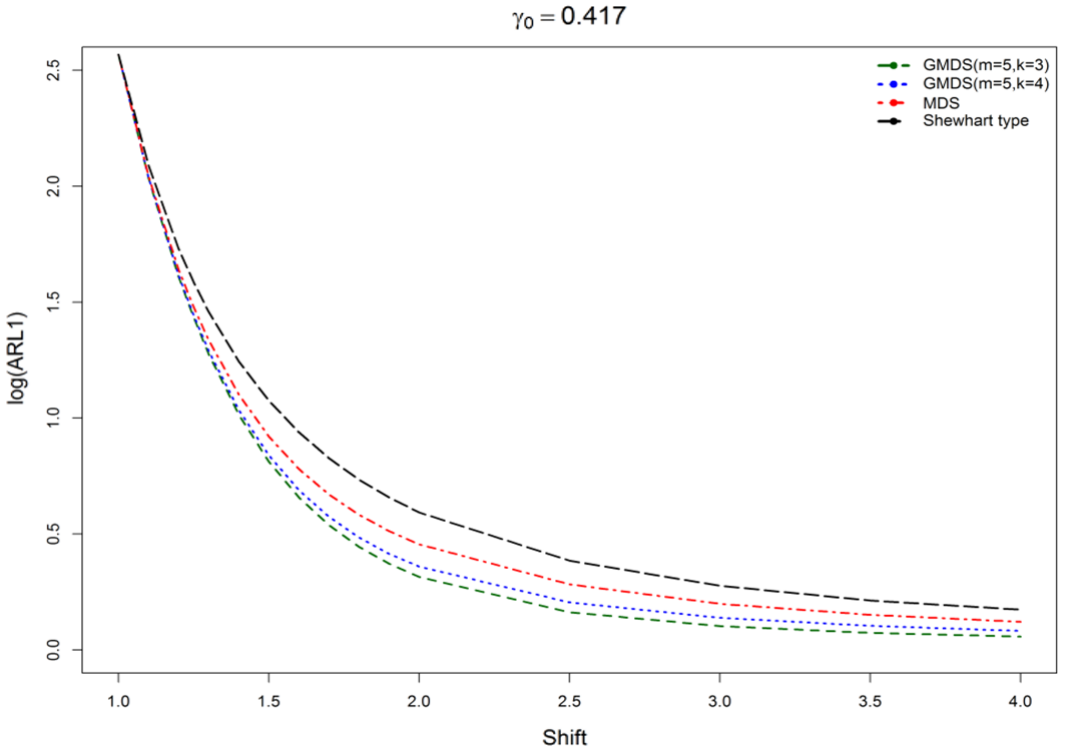
ARL curves of $\gamma$-control chart for three charts at *n* = 5, *m* = 5, $\gamma_{0}$ = 0.417, ARL_0_ = 370 for real data.

**Figure 4:**
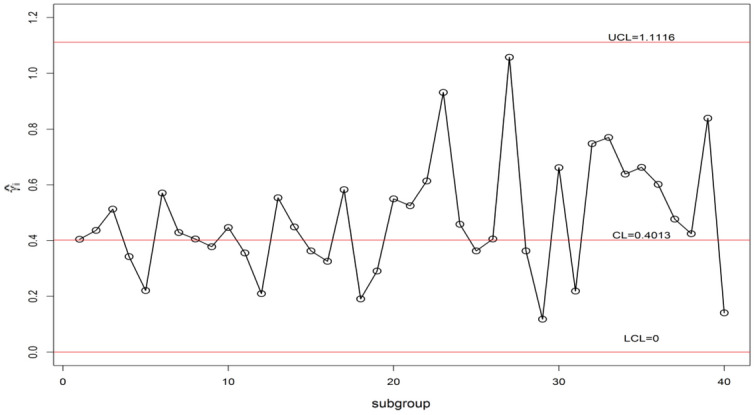
Shewhart type $\gamma$-control chart for sintering process data.

**Figure 5:**
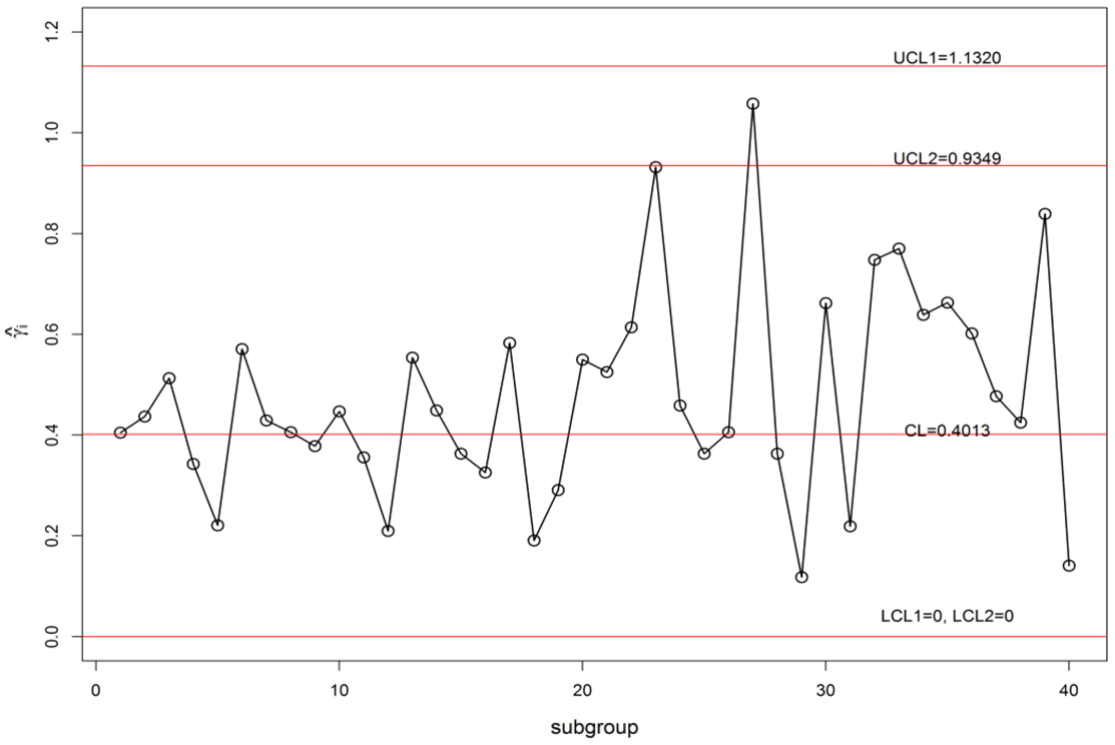
$\gamma$-control chart using MDS sampling for sintering process data.

**Figure 6:**
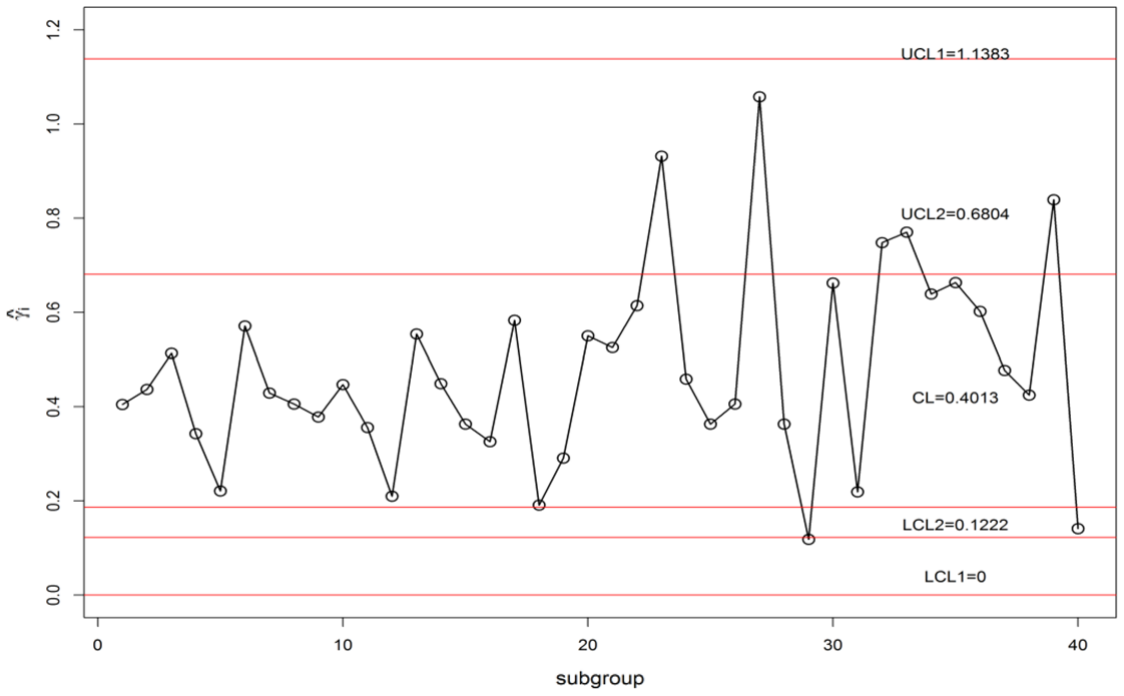
$\gamma$-control chart using GMDS sampling for sintering process data.

We display the planned $\gamma$-control chart attitude by an actual data set calmed as of a sintering process manufacturing mechanical parts. The detailed description of the data set can be found in supervising the CV using EWMA Charts studied by^9^. They have collected the under Phase-I dataset for 20 subgroups of each size 5 (i.e. *n* = 5). The analysis of the in-control data gives an estimate $\gamma_{0}$ = 0.417. The chart constants for proposed $\gamma$-control chart for GMDS, MDS, and Shewhart type at n = 5, $\gamma_{0}$ = 0.417 along with out-of-control ARL (ARL_1_) are presented in Table 8. Furthermore, the accomplishment of the planned $\gamma$-control chart is portrayed in Fig. 3. From Eqs. (4) and (5), we get $\mu_{0} \left( {\hat{\gamma }} \right)$ = 0.4013 and $\sigma_{0} \left( {\hat{\gamma }} \right)$ = 0.1733. Using the chart constant in Table 8 at *n* = 5, *m* = 5 planned $\gamma$-control chart limits of GMDS are UCL_1_ = 1.1383, UCL_2_ = 0.6804, LCL_2_ = 0.1222 and LCL_1_ = 0; for MDS are UCL_1_ = 1.1320, UCL_2_ = 0.9349, LCL_2_ = 0 and LCL_1_ = 0; for Shewhart type are UCL = 1.1116 and LCL = 0. The Phase-II dataset also borrowed from^9^. These data consists of 20 new subgroups of each size 5 taken from the process when it is shifted to 1.25, which give as $\gamma_{1} = c\gamma_{0} = 1.25 \times 0.417 = 0.521$. The chart statistics $\hat{\gamma }_{i}$ and control limits for three $\gamma$-control charts are plotted in Figs. 4, 5 and 6 for comparison of the charts. The designed control chart for this example it is possible to illustrate as: the process can be declared as in-control if 5 previous values of $\hat{\gamma }_{i}$ lie within the interior control limits of the planned $\gamma$-control chart with reference to MDS plan. Whereas, in the case of planned $\gamma$-control chart under GMDS plan, the process is believed as under control if at least 4 out of 5 previous values of $\hat{\gamma }_{i}$ lie within the interior control limits.

**Table 8:** ARLs of real data for $\gamma$-control chart for GMDSS when *n* = 5, $\gamma_{0}$ = 0.417, ARL_0_ = 370.

*c*	GMDS	MDS	Shewhart type
	$k_{1}$ = 4.2530	$k_{1}$ = 4.2165	
	$k_{2}$ = 1.6105	$k_{2}$ = 3.0795	L = 4.0992
1.00	370.02	370.13	370.01
1.10	107.97	110.07	122.85
1.20	40.72	43.80	53.88
1.25	27.07	30.08	38.53
1.30	18.86	21.65	28.69
1.40	10.34	12.62	17.54
1.50	6.50	8.32	11.87
1.60	4.54	6.02	8.67
1.70	3.45	4.68	6.71
1.80	2.78	3.82	5.43
1.90	2.36	3.25	4.55
2.00	2.07	2.85	3.92
2.50	1.45	1.92	2.43
3.00	1.27	1.58	1.89
3.50	1.18	1.42	1.63
4.00	1.14	1.32	1.49

From Fig. 4, it is noticed that all sample points within control limits, which suggests as Shewhart type $\gamma$-control chart unable to identify change. Whereas, the proposed $\gamma$-control chart with reference to the MDS plan not able to identify shift since 5 previous values of $\hat{\gamma }_{i}$ recline in the inner control limits. On the other hand, the $\gamma$-control chart with reference to the GMDS plan trips the out-of-control signs at subgroups sizes 32 and 33, respectively (see Fig. 6). Hence, from discussion obviously demonstrates that the planned $\gamma$-control chart with reference to GMDS plan is more perceptive in discovering process change as examined to the $\gamma$-control chart with reference to the MDS and Shewhart type plans. Therefore, from this industrial application the $\gamma$-control chart using GMDS endures better quality amongst considered control charts for the rapid revealing of the process change.

### Industrial application-2

**Figure 7:**
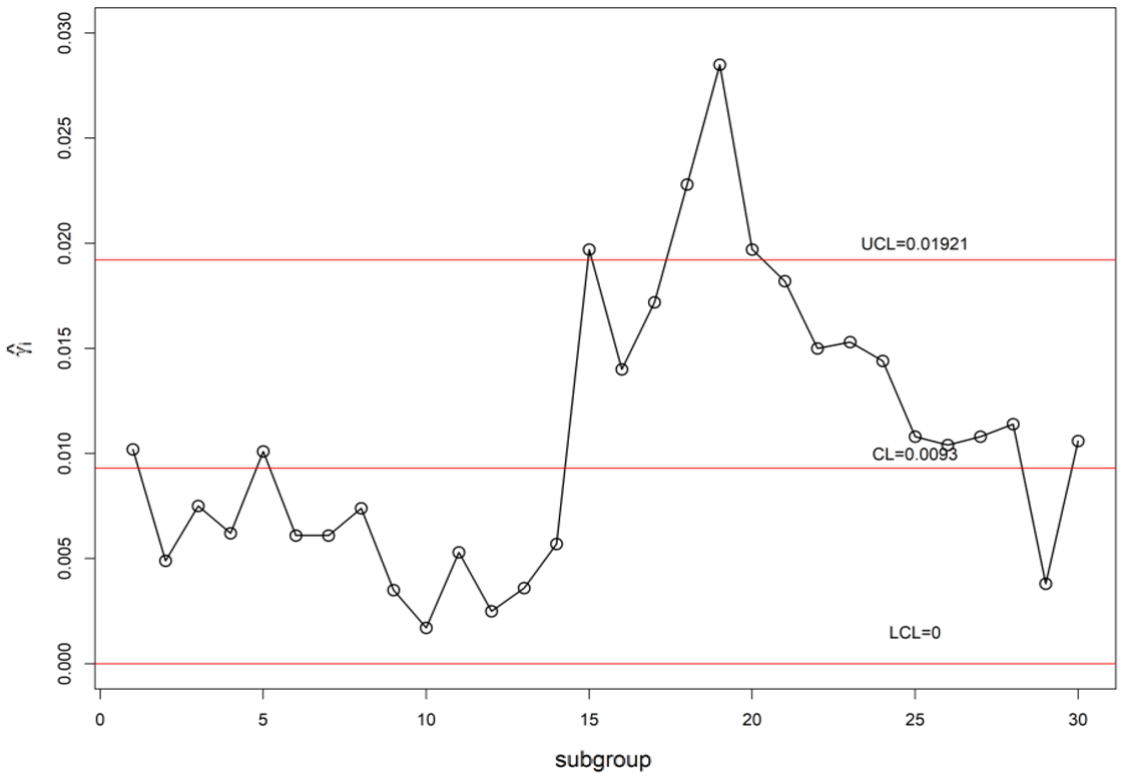
Shewhart type $\gamma$-chart for die casting hot chamber process data.

**Figure 8:**
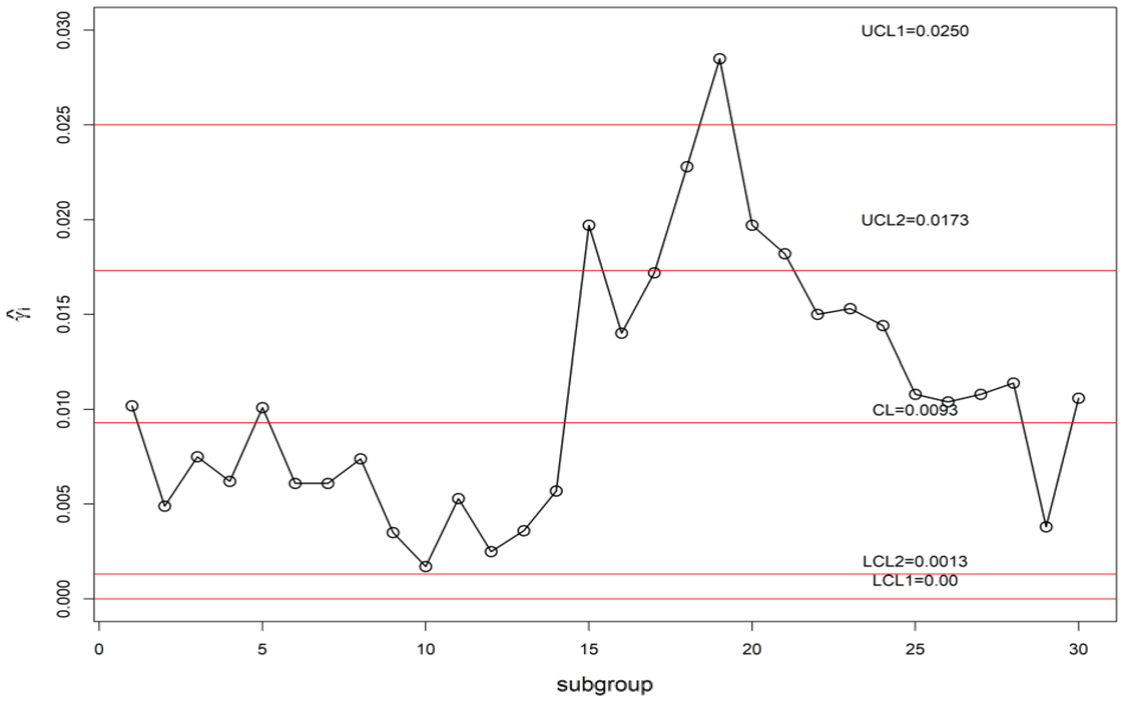
$\gamma$-chart using MDS sampling for die casting hot chamber process data.

**Figure 9:**
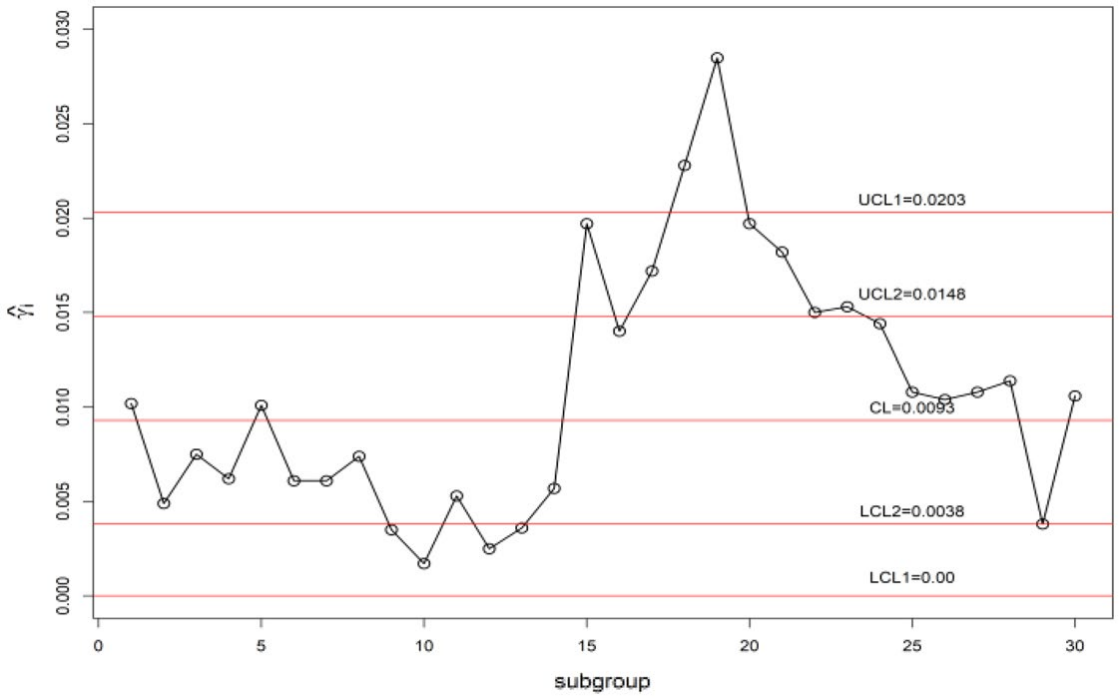
$\gamma$-chart using GMDS sampling for die casting hot chamber process data.

We display the planned $\gamma$-control chart attitude by an illustrated with real industrial data of a die casting hot chamber process. These data are provided by a zinc-alloy (ZAMAK) manufacturing company in Tunisia and introduced in monitoring the coefficient of variation using control charts with run rules studied by^52^. They have collected the under Phase-I dataset of 30 samples, each having 5 observations are accumulated. By adopting this method, the estimated $\gamma_{0}$ is computed from the Phase-I dataset as $\gamma_{0}$ = 0.01. The chart constants for proposed $\gamma$-control chart for GMDS, MDS, and Shewhart type at n = 5, $\gamma_{0}$ = 0.01 along with out-of-control ARL (ARL_1_) are presented in Table 9. Furthermore, the accomplishment of the planned $\gamma$-control chart is portrayed in Fig. 7. From Eqs. (4) and (5), we get $\mu_{0} \left( {\hat{\gamma }} \right)$ = 0.009255 and $\sigma_{0} \left( {\hat{\gamma }} \right)$ = 0.00341. Using the chart constant in Table 9 at *n* = 5, *m* = 5 planned $\gamma$-control chart limits of GMDS are UCL_1_ = 0.0203, UCL_2_ = 0.0148, LCL_2_ = 0.0038 and LCL_1_ = 0; for MDS are UCL_1_ = 0.02501, UCL_2_ = 0.0173, LCL_2_ = 0.0013 and LCL_1_ = 0; for Shewhart type are UCL = 0.01921 and LCL = 0. The Phase-II dataset also borrowed from^52^. These data consists of 30 new subgroups of each size 5 taken from the process when it is shifted to 1.20, which give as $\gamma_{1} = c\gamma_{0} = 1.20 \times 0.01 = 0.012$. The chart statistics $\hat{\gamma }_{i}$ and control limits for three $\gamma$-control charts are plotted in Figs. 7, 8 and 9 for comparison of the charts. The designed control chart for this example it is possible to illustrate as: the process can be declared as in-control if 5 previous values of $\hat{\gamma }_{i}$ lie within the interior control limits of the planned $\gamma$-control chart with reference to MDS plan. Whereas, in the case of planned $\gamma$-control chart under GMDS plan, the process is believed as under control if at least 3 out of 5 previous values of $\hat{\gamma }_{i}$ lie within the interior control limits. From Fig. 7, it is noticed that process is out-of-control for the Shewhart type $\gamma$-control chart. Whereas, the developed $\gamma$-control chart based on the MDS plan fail to identify shift since 5 previous values of $\hat{\gamma }_{i}$ lie in the inner control limits (see Fig. 8). On the other hand, the $\gamma$-control chart with reference to the GMDS plan trips the out-of-control signs at subgroups sizes 19 (see Fig. 9). Hence, from discussion obviously demonstrates that the planned $\gamma$-control chart with reference to GMDS plan is more perceptive in discovering process change as examined to the $\gamma$-control chart with reference to the MDS and Shewhart type plans. Therefore, from this industrial application the $\gamma$-control chart using GMDS endures better quality amongst considered control charts for the rapid revealing of the process change.

**Table 9:** ARLs of real data for $\gamma$-control chart for GMDSS when *n* = 5, $\gamma_{0}$ = 0.01, ARL_0_ = 370.

*c*	GMDS	MDS	Shewhart type
	$k_{1}$ = 3.2430	$k_{1}$ = 4.6145	
	$k_{2}$ = 1.610	$k_{2}$ = 2.343	L = 2.9176
1.00	370.09	370.15	370.41
1.10	86.09	98.28	90.41
1.20	26.17	30.53	33.02
1.25	10.73	12.66	22.23
1.30	5.64	6.74	15.82
1.40	3.58	4.32	9.14
1.50	2.60	3.15	6.03
1.60	2.07	2.52	4.38
1.70	1.76	2.13	3.42
1.80	1.57	1.88	2.80
1.90	1.45	1.71	2.40
2.00	1.18	1.31	2.11
2.50	1.10	1.17	1.46
3.00	1.07	1.11	1.25
3.50	1.06	1.09	1.16
4.00	1.05	1.08	1.11

### Simulation investigation

In order to investigate the implementation of the planned control chart over the available control charts, a simulation study is conducted. In this investigation, generated 40 samples of each size 5 from normal distribution. The first 20 subgroups of each size 5 are creating from normal population by mean 10 and in-control SD,$\sigma = \gamma_{0} \mu = 0.2 \times 10 = 2.0$ = 2.0 and last 20 subgroups of each size 5 are creating from normal population with mean 10 and out-of-control SD $\sigma_{1} = \gamma_{1} \mu = c\gamma_{0} \mu = 1.3 \times 0.20 \times 10 = 2.6$. That is, the process CV is shifted after 20 subgroups with a CV shift of *c* = 1.3. The data is reported in Table 10 along with computed sample CV, $\hat{\gamma }_{i} = \frac{{S_{i} }}{{\overline{X}_{i} }}$ for each subgroup. To judge the speedy revealing talent of the designed $\gamma$-control charts using GMDS as against the MDS and Shewhart type control charts at *n* = 5, *m* = 5, $\gamma_{0}$ = 0.20 and ARL_0_ = 370.

**Table 10:** The simulated data when *n* = 5, $\gamma_{0}$ = 0.2, ARL_0_ = 370.

Subgroup No	Sample					$\overline{x}_{i}$	$S_{i}$	$\hat{\gamma }_{i}$
	1	2	3	4	5			
1	9.6727	7.6509	12.0098	5.5648	8.8716	8.7540	2.3899	0.2730
2	8.9740	11.0280	11.1216	7.6539	12.8959	10.3347	2.0432	0.1977
3	12.7649	7.2960	12.4456	11.4314	10.3261	10.8528	2.2055	0.2032
4	9.6017	9.6980	8.6483	9.3515	11.7032	9.8005	1.1401	0.1163
5	11.7929	9.3517	7.9097	11.0388	9.8542	9.9894	1.5086	0.1510
6	9.2248	9.9627	10.4372	11.7108	8.1071	9.8885	1.3455	0.1361
7	9.7516	12.1157	7.5942	10.4880	9.9018	9.9702	1.6255	0.1630
8	7.9812	12.3240	11.5248	8.5531	14.5234	10.9813	2.7176	0.2475
9	14.7005	10.5213	9.8021	11.4598	9.4106	11.1789	2.1175	0.1894
10	9.6824	10.6263	9.3941	9.6439	7.5157	9.3725	1.1391	0.1215
11	11.6834	10.6718	8.5494	8.8757	5.4059	9.0372	2.4044	0.2661
12	8.9716	10.4594	7.7741	6.5185	9.9192	8.7285	1.6022	0.1836
13	12.9957	10.3892	8.5864	11.8228	9.6585	10.6905	1.7449	0.1632
14	9.6438	11.5588	5.0091	9.4906	8.4138	8.8232	2.4144	0.2736
15	7.2588	11.4295	12.3630	11.4522	12.7163	11.0440	2.1897	0.1983
16	11.8809	14.1348	9.1609	11.2139	12.5001	11.7781	1.8203	0.1545
17	6.4436	13.4869	9.1689	10.3478	12.3099	10.3514	2.7539	0.2660
18	12.1015	9.1800	8.5581	8.8727	11.5145	10.0454	1.6372	0.1630
19	6.6813	10.6858	10.9172	8.3641	10.6927	9.4682	1.8753	0.1981
20	8.7884	8.4312	9.9928	4.1544	9.9188	8.2571	2.3937	0.2899
21	14.0224	9.7283	8.4227	10.2967	8.0815	10.1103	2.3690	0.2343
22	6.8309	4.0828	10.0778	11.9865	6.0444	7.8045	3.1841	0.4080
23	13.2513	9.1042	10.9809	8.7726	11.2084	10.6635	1.8098	0.1697
24	9.8558	7.4853	11.7181	9.8224	11.0632	9.9890	1.6169	0.1619
25	13.0452	6.7631	12.5194	9.8035	8.3609	10.0984	2.6821	0.2656
26	10.3525	8.3959	9.6606	9.9372	3.5681	8.3829	2.7888	0.3327
27	12.0828	13.0944	12.5229	11.7187	8.8568	11.6551	1.6464	0.1413
28	8.8916	10.2671	9.9391	13.6299	7.3723	10.0200	2.3121	0.2307
29	10.6660	6.7441	12.6226	13.5254	6.1660	9.9448	3.3554	0.3374
30	11.1429	11.9908	13.0395	9.7210	9.9652	11.1719	1.3893	0.1244
31	14.2716	13.6499	7.3951	8.0448	9.1092	10.4941	3.2307	0.3079
32	10.7156	9.9047	5.5130	8.4744	5.0385	7.9292	2.5573	0.3225
33	11.3826	10.6295	10.4531	5.4940	8.0536	9.2025	2.4200	0.2630
34	19.8739	9.7463	13.1626	10.9796	8.6304	12.4786	4.4632	0.3577
35	7.3668	8.0607	8.7967	12.6663	10.5499	9.4881	2.1356	0.2251
36	9.1158	8.7666	15.9905	11.3469	10.1110	11.0662	2.9297	0.2647
37	12.8522	7.0179	8.7690	11.9335	12.8466	10.6838	2.6480	0.2478
38	11.3589	12.7938	11.2923	12.7306	10.8812	11.8113	0.8873	0.0751
39	3.3446	7.6954	11.6239	10.9950	8.0947	8.3507	3.2887	0.3938
40	6.1460	9.2832	6.4222	8.1492	11.4696	8.2941	2.1910	0.2642

**Figure 10:**
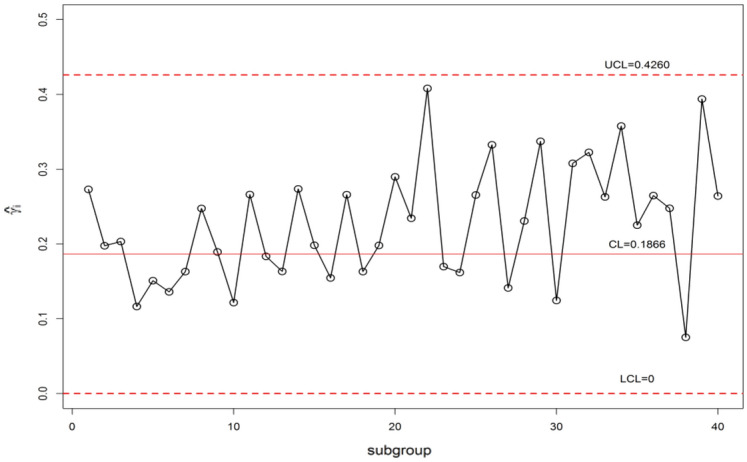
$\gamma$-control chart for Shewhart type sampling simulated data when *n* = 5, $\gamma_{0}$ = 0.20, ARL_0_ = 370.

**Figure 11:**
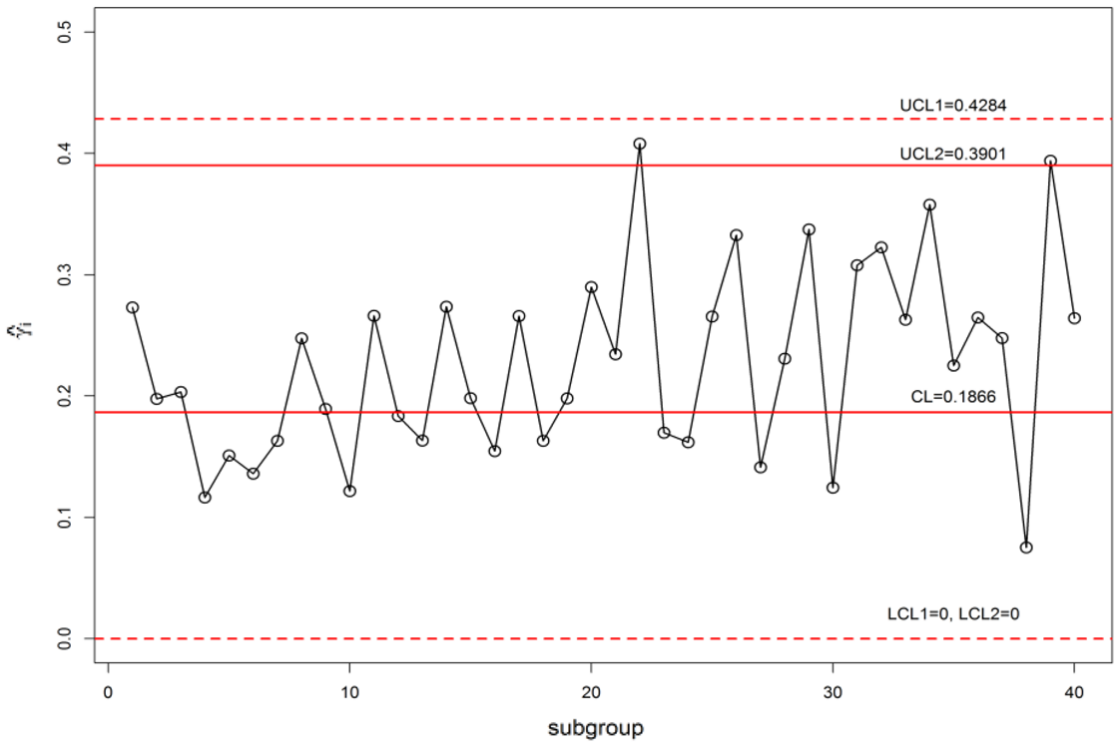
$\gamma$-control chart using MDS sampling simulated data when *n* = 5, $\gamma_{0}$ = 0.20, ARL_0_ = 370.

**Figure 12:**
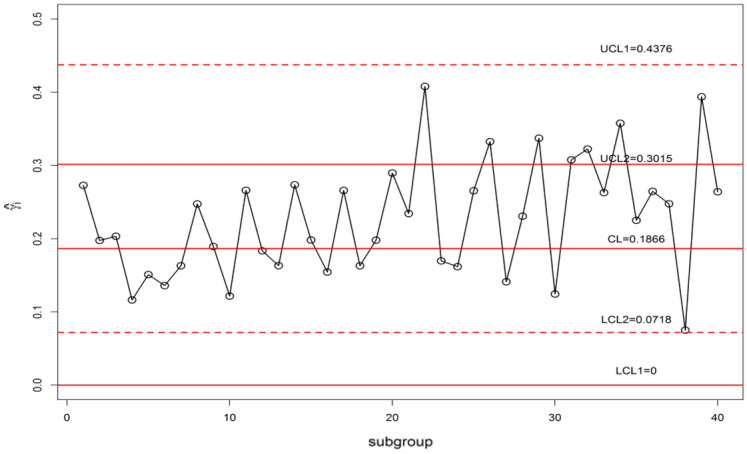
$\gamma$-control chart using GMDS sampling simulated data when *n* = 5, $\gamma_{0}$ = 0.20, ARL_0_ = 370.

The control chart constants for *n* = 5, *m* = 5, $\gamma_{0}$ = 0.20 and ARL_0_ = 370 are available in Table 10. The Shewhart type $\gamma$-control charts are given in Fig. 10 and $\gamma$-control charts using MDS sampling with *m* = 5, *k* = 5 is depicted in Fig. 11. The $\gamma$-control charts under GMDS sampling at values m = 5, *k* = 3 is displayed in Fig. 12. In MDS scheme using $\gamma$-control charts, the process is supposed to be under control provided that the previous 5 (since *m* = 5) $\hat{\gamma }_{i}$ values within the inner control limits whereas in case of GMDS under $\gamma$-control charts, the process is affirmed as in-control if no less than 3 out of 5 previous $\hat{\gamma }_{i}$ values between the interior control limits.

It is clear that from Figs. 10 and 11, the $\gamma$-control charts under Shewhart type and MDS designs are failed to detect the shift. Whereas, when we observe Fig. 12 it can be detected that using $\gamma$-control charts under GMDS scheme signals out at subgroup numbers 34 ( since in the preceding 5 statistics 2 only within the inner control limits). This simulation investigates that the planned $\gamma$-control chart under GMDS sampling is to a greater extent perceptive to detect the change of manufactured output over the accessible $\gamma$-control charts with reference to MDS and under Shewhart type scheme.

## Conclusions

This paper aimed to develop a coefficient of variation (CV) control chart based on the generalized multiple dependent state (GMDS) sampling method for CV detection. We conducted a thorough examination of this designed control chart in comparison to control charts based on multiple dependent state sampling (MDS) and the Shewhart-type CV control chart, with a focus on average run lengths. The results were then compared to two control charts available in the existing literature. We further illustrated the implementation of the proposed control chart through concrete examples and a simulation study. The findings clearly indicated that the GMDS sampling control chart outperforms the MDS sampling control chart significantly in detecting process shifts. Consequently, the control chart approach introduced in this paper holds great promise for applications in textile and medical industries, especially when researchers seek to identify small to moderate shifts in the CV. This research has provided valuable insights into process control and quality monitoring, offering a robust tool for improving product quality and operational efficiency in these sectors. This paper also studied the planned $\gamma$-control chart using GMDS sampling over the MDS, Shewhart type $\gamma$-control charts, SH-$\gamma$ chart and $RR_{2,3} - \gamma \,{\text{chart}}$ with respect to the ARLs. The planned chart is moreover imposed for real example and also using a simulation study and it shows that design $\gamma$-control chart using GMDS sampling detected out-of-control samples over the MDS and Shewhart type $\gamma$-control charts. The proposed control chart performed well as compared with the existing SH-$\gamma$ chart developed by^6^, the 2-out-of-3 Run Rules (denoted as $RR_{2,3} - \gamma \,{\text{chart}}$) suggested by^5^ control charts. The results indicate that the GMDS sampling control chart is a great extent accurate than the MDS sampling control chart to differentiating the process shift. The designed control chart approach in this paper can be employed in textile industrial and medical environments specifically when the experimenter would like to discover a small and moderate shift in the CV. The proposed control charts by means of a cost form is an area for future research. This research can be studied for sampling plans for both normal and skewed distribution as future research. The potential for future research lies in considering the application of the proposed chart incorporating EWMA and CUSUM statistics.

## Data Availability

The datasets used and/or analysed during the current study available from the corresponding author on reasonable request.
